# Volatile Compounds, Odour and Flavour Attributes of Lamb Meat from the Navarra Breed as Affected by Ageing

**DOI:** 10.3390/foods10030493

**Published:** 2021-02-25

**Authors:** Kizkitza Insausti, María T. Murillo-Arbizu, Olaia Urrutia, José A. Mendizabal, María J. Beriain, Michael J. Colle, Phillip D. Bass, Ana Arana

**Affiliations:** 1 IS-FOOD, School of Agricultural Engineering and Biosciences, Public University of Navarra (UPNA), 31006 Pamplona, Navarra, Spain; mariateresa.murillo@unavarra.es (M.T.M.-A.); olaia.urrutia@unavarra.es (O.U.); jamendi@unavarra.es (J.A.M.); mjberiain@unavarra.es (M.J.B.); aarana@unavarra.es (A.A.); 2 Department of Animal and Veterinary Science, University of Idaho, Moscow, ID 83844, USA; mjcolle@uidaho.edu (M.J.C.); pbass@uidaho.edu (P.D.B.)

**Keywords:** lamb, volatile compounds, odour and flavour, ageing

## Abstract

This study aimed to assess the influence of ageing on the volatile compounds, as well as odour and flavour attributes of lamb meat from the Navarra breed. Twenty-one male lambs were fed a commercial concentrate diet after weaning and were harvested at 101 ± 6.5 days of age. From the Longissimus thoracis, 26 volatile compounds were identified, with hexanal, 2-propanone, and nonanal the most abundant (57.17% relative percentage abundance, RPA). The effect of ageing (1 vs. 4 d) was observed (*p* < 0.05) in six compounds: 1,4-dimethylbenzene decreased with ageing, while tridecane, 3-methylbutanal, 2-heptanone, 3-octanone, and 1-octen-3-ol increased. In general, ageing was linked to a decrease in livery and bloody flavour, bloody odour and ethanal, and an increase in pentane, hexanal, and heptanal, which are usually associated with fresh green grass and fat descriptors. Consequently, ageing lamb from the Navarra breed for four days might have a positive effect on meat sensory odour and flavour quality.

## 1. Introduction

Among meat quality parameters, aroma is the sensory attribute of cooked meat that most strongly contributes to the identification of the animal species, especially in lamb [1]. Indeed, flavour was deemed as the attribute that most influenced the overall acceptability of lamb meat in a study carried out with Spanish consumers [2].

Breed, together with live weight, carcass weight, and precocity, is considered one of the most important meat quality factors affecting odour and flavour [3]. However, the complex interaction among them makes it difficult to compare the results of different studies. These particular characteristics may have the potential to increase market appeal through specific branding identification and labelling [4,5]. In this sense, lamb from the Navarra breed is a regionally known breed in Spain that represents the main source of lamb meat to the market of this Spanish region. It is commonly marketed under the Protected Geographical Indication (PGI) Cordero de Navarra/Nafarroako Arkumea [6] (*Boletín Oficial del Estado* (BOE) 140, 12/06/2002), which means that they need to be born, raised and slaughtered in Navarra in order to be marketed under the PGI requirements. So far, very little research on the quality of meat from these specific animals has been published [7]. Besides, there has not been a study published on the composition of volatile compounds and the effect that ageing may have in differentiating the product of the Navarra breed from other PGI lamb products in Europe.

Regarding post-harvest factors that affect meat quality, ageing is a post-mortem process where a complex set of reactions take place, which, among many things, include flavour profile [8]. The effect of ageing has been previously reviewed in beef volatile compounds [9,10]. However, little is known in lamb about the effect of ageing on volatile compounds and its contribution to sensory quality, and especially concerning characteristic odour and flavour. In Spain suckling lambs are often consumed soon after slaughter (even after 24 h post-mortem), and it is traditionally assumed that no significant changes in the abovementioned parameters occur during ageing.

Previous research has studied heavy lamb carcass meat quality from animals produced in different countries such as Australia [11]. However, in Southern Europe, the most common lamb commercial types are suckling lambs (<7 kg carcass weight) and light weight lambs (10–13 kg carcass weight) [12], which are milder in odour and flavour. On the contrary, the research on suckling and light lambs is limited.

Finally, associating sensory analysis with gas chromatography data is difficult. Few studies have quantified the contribution of these compounds to lamb flavour. Bueno et al. [13] using partial-least square models, reported that lamb flavour is the result of a complex balance among different odour-active volatile compounds, but they did not study the effect of ageing.

The objective of the current study was to test the hypothesis that ageing would affect the volatile compounds, as well as odour and flavour attributes of lamb meat from the Navarra breed.

## 2. Materials and Methods

Animal care, handling and experimental procedures were in compliance with relevant international guidelines (European Union procedures on animal experimentation—Directive 2010/63/EU) that regulate the protection of animals used for scientific purposes [14]. Lambs were slaughtered at a commercial abattoir in Pamplona (Spain) in accordance with the Council Regulation, EC, No. 1099 (2009) [15] concerning the protection of animals at slaughter.

### 2.1. Animal Handling and Sampling

A total of 21 unrelated male lambs of the Navarra breed were studied. Lambs were raised with their mothers from birth until weaning (53 ± 3 days of age). After weaning, lambs were introduced to a commercially available pelletised concentrate ration (% dry matter (DM): barley 15.2, soya 11.8, maize 10.0, calcium carbonate (CaCO_3_) 2.2, mineral–vitamins supplement 0.6) (94.4% organic matter, OM; 16.0% crude protein, CP; 2.18% ether extract; 5.7% crude fibre; and 2.70 Mcal of metabolisable energy, ME/kg of DM) and barley straw (cut into lengths of 5 cm). All feed was fed ad libitum until slaughter (27.8 ± 0.78 kg live weight (LW), and 101 ± 6.5 days of age; average time on feed was 40 days).

Twenty-four hours after slaughter, the Longissimus thoracis (LT) muscle was removed from the left side of each carcass and cut into four pieces. LT muscles were approximately 30 cm long, and the four subsamples were consistent in thickness and size. Two of the four subsamples were immediately vacuum packaged and frozen at −18 °C (1 day of ageing). Before freezing, samples were vacuum packaged in polyamide/polyethylene (PA/PE) pouches (Vaessen Schoemaker Ind., Barcelona, Spain, 120 µm thick with an (oxygen) O_2_ permeability of 1 cc/m^2^/24 h, a CO_2_ permeability of 3 cc/m^2^/24 h, an N_2_ (nitrogen) permeability of 0.5 cc/m^2^/24 h measured at 5 °C and 75% relative humidity (RH) and a water vapour transmission rate of 3 g/m^2^/24 h at 38 °C and 100% RH). Vacuum packaging (99% vacuum) was performed using a LERICA model C412 machine (San Giovanni Lupatoto, Italy).

The remaining two pieces of Longissimus thoracis were aged in the dark until day 4 post-mortem in commercial polystyrene foam meat trays over-wrapped with a commercial transparent film food at 4 ± 1 °C. After ageing (day 4), the steaks were vacuum packaged and frozen at −18 °C until subsequent analysis. Half of the samples aged 1 and 4 days were used for sensory analysis and the other half were used for volatile compound analysis. Samples were defrosted for 24 hours at 4 °C prior to analysis.

### 2.2. Volatile Compound Analysis of Muscle

The procedure for volatile compound analysis was previously reported in Insausti et al. [16].

The experimental units were the individual animals (*n* = 21) and each analysis was carried out in duplicate. Briefly, the steaks were thawed at 4 °C overnight. Following thawing, steaks were over-wrapped in aluminium foil and cooked at 200 °C in an electric double plate grill (model Panini Medio, Silanos, Barcelona, Spain) to an internal temperature of 70 °C [17]. Internal temperature was monitored at the centre of the steaks using a Digitron model 3246 thermocouple (Hereford, UK). After cooking, the steaks were trimmed of fat and visible connective tissue, and 10 g of ground cooked lamb were placed immediately in a headspace vial (Pyrex^®^ Corning 7150-25 Sparger Fritless 25 mL). The vial was attached to a purge-and-trap sample concentrator (Tekamr-Dohrmann 3100 Sample Concentrator, OH, USA) that included an external heater that was set to 70 °C. Then, the sample was purged for 20 min at a helium flow rate of 40 mL/min. The headspace volatiles were collected on a Tenax GC trap (60/80 mesh) at 15 °C and thermally desorbed at 225 °C for 2 min at a helium flow rate of 40 mL/min. Before each extraction the Tenax GC trap was conditioned at 230 °C for 35 min at a helium carrier flow rate of 40 mL/min.

The desorption unit was connected to GC-MS equipment (Gas chromatography–mass spectrometry), so volatile compounds were directly transferred (splitless mode) to the column. Identification and quantification of the volatile compounds was performed on an HP-6890 gas chromatograph (Hewlett-Packard, Madrid, Spain) connected to an HP-5973 quadrupole mass spectrometer (Hewlett-Packard, Madrid, Spain), using an HP-5 capillary column (5% phenyl methyl siloxane; 50 m × 320 µm i.d. × 1.05 µm film thickness) (Hewlett-Packard, Madrid, Spain) with helium as the carrier gas. The column temperature programme started out at 35 °C for 15 min and then rose at a rate of 8 °C/min to 220 °C, where it was held for 5 min. Injector temperature was set at 250 °C. Desorbed volatile compounds were injected in split mode at a column flow rate of 1.5 mL/min, a column head pressure of 6.0 psi, and a split ratio of 5:1. The main mass spectrometer operational settings were ionisation voltage, 70 eV; ion source temperature, 230 °C; electron multiplier voltage, 2000 V; scan speed, 3.32 scan/s; scan range, 30 to 250 amu (atomic mass unit); and quadrupole temperature, 108 °C.

Tentative identification was based on the comparison of their mass spectra with spectra from known compounds through a computerised search of the mass spectra Wiley 275 library. To confirm the identification, Kovats Linear Retention Indexes (LRIs) were calculated. The LRIs were obtained by previous injection of standards of n-alkanes (C6-C24, Sigma-Aldrich, Saint Louis, MO, USA). Results were expressed in arbitrary area units (peak area) ×10^5^, following Fruet et al. [18].

### 2.3. Sensory Analysis

Quantitative descriptive analysis [19] was used to assess lamb odour and flavour. To do so, a descriptive panel (*n* = 7) was selected and trained (ISO 8586-1:1993, International Organization for Standardization) and adapted by Gorraiz et al. [20] for meat from young ruminants, evaluated odour and flavour of lamb samples.

Meat cuts were thawed at 4 °C overnight and cooked at 200 °C in a double plate grill (model Panini Medio, Silanos, Barcelona, Spain), to an internal temperature of 70 °C [17]. Internal temperature was monitored at the centre of the steaks using a probe Digitron (Hereford, UK) model 3246 thermocouple. 

The cooked meat cuts were immediately cut into cubes of 2 cm × 1.5 cm × 1.5 cm, they were wrapped with aluminium foil, previously coded with a random three-digit code, and they were kept warm at 60 °C before being served to the panellists no later than 15 min after cooking. Panellists were provided with slices of apple and water for cleansing their palate between samples. In each session, six samples were assessed using a balanced and randomised design. Sessions were carried out in individual booths (designed according to ISO 8589:2007) under red lighting in order to avoid stimulus error and to mask any meat colour differences. Each panellist was served each sample individually in a different order to avoid the carry-over effect. Attributes evaluated included: characteristic lamb odour, bloody odour, livery odour, characteristic lamb flavour, bloody flavour, livery flavour, fatty flavour, and aftertaste flavour, using a 150-mm unstructured hedonic scale, with a mark 10 mm from the left end, representing “low intensity” and 10 mm from the right end, representing “high intensity”. The results were quantified by measuring the distance in millimetres of the panellists’ mark from the left end.

### 2.4. Data Processing and Statistical Analysis

All data were analysed using IBM SPSS Statistics 25 for Windows (SPSS Inc. Corporation, NY, USA) software. A one-way analysis of variance with ageing duration as the fixed effect was carried out. For the sensory data, the sensory analysis session and panellists were considered as random effects (Equation (1)).

(1) $$y_{\mathrm{ijkl}}\;=\;µ\;+\;A_{i}\;+\;p_{j}\;\times \;s_{k}\;+\;e_{\mathrm{ijkl}}$$

where A_i_ is the fixed factor “ageing”, and P_j_ and S_k_ are the random effects assigned to panellists (P) and session (S). Statistical difference was declared at *p* = 0.05.

Associations between volatile compounds and sensory attributes were also investigated using simple correlation Pearson coefficients. The factorial analysis, using the principal component extraction (PCA) and Varimax rotation method [21], was applied to study the relationship between the sensory attributes on one hand, the volatile compounds on the other hand, and finally the relationship between sensory and volatile compounds. The discriminant analysis was carried out to try and find out which of the studied variables, both sensory and volatile compounds, contributed more to the separation among days of storage. 

## 3. Results and Discussion

### 3.1. Profile of Lamb Meat Volatile Compounds

A total of 26 volatile compounds with different chemical characteristics were identified in the Longissimus thoracis muscle of lambs from the Navarra breed (Table 1).

Part of the volatile compounds found in cooked meat come from lipid oxidation reactions, while some others come from the Maillard reaction [28]. The former include straight chain aldehydes, ketones, hydrocarbons, alcohols, and alkyfurans. The later are formed via Maillard reaction and include heterocyclic nitrogen and sulphur compounds, such as pyrazines, thiphenes, and thiazoles, as well as furanones and furfurals [28]. Non-heterocyclic compounds, such as Strecker aldehydes, alkadiones and hydroxyketones are also produced in the Maillard reaction, as well as aliphatic and furan disulfides.

Most of the volatile compounds obtained in lamb meat (Navarra breed) in the current study, regarding both number and peak area values, were aliphatic hydrocarbons (*n* = 6 and total relative percentage abundance (RPA) = 11.27%), aliphatic aldehydes (*n* = 9 and total RPA = 54.86%) and aliphatic ketones (*n* = 4 and total RPA = 18.66%). Thus, a high level of lipid-derived volatile compounds, with an 84.79% RPA, was obtained, representing 19 out of the total 26 identified compounds.

The greater occurrence of aldehydes in comparison with other classes of volatile compounds is, in general, representative of the volatile profile of ruminant meat [29] and could be related to a high level of concentrate in the diet. Indeed, hexanal, nonanal and heptanal (Table 1) were the aldehydes mostly derived from the oxidation of the polyunsaturated fatty acids (PUFAs) during cooking [3] and their level tends to increase with concentrate-based diets that are characterised by a high content in linoleic acid and low amounts of naturally occurring antioxidants [30,31].

Considering individual volatile compounds, those with high RPA values were pentane, ethanal, hexanal, nonanal, 2-propanone, carbon disulphide, and 1-octen-3-ol with values ranging from 4 to 31%. According to the Flavournet database, these compounds are said to provide grass and fat flavour (hexanal and nonanal), pungent (ethanal), cabbage (carbon disulphide) and mushroom (1-octen-3-ol).

Hexanal is the volatile compound with the greatest presence identified with an RPA of 31.31% (Table 1), and it is known to derive from oxidation of linoleic acid in muscle (C18:2*n*–6) [32], probably its presence is due to the finishing period of animals on concentrate [33]. It has a high aromatic potential with notes of green grass [24] and with only traces, it already contributes to flavour [34].

The second most abundant volatile compound obtained in the samples was 2-propanone having a RPA of 15.59% (Table 1). This family of volatile compounds (aliphatic ketones) seems to originate following lipid oxidation [28]. They have very low detection thresholds, and they seem to contribute to the fatty flavour of cooked meat [26]. 2-propanone is a frequently reported volatile compound in meat, even reaching a 71% of RPA in beef [35]. In this study however, in lamb, it only reached a 15.59% RPA.

Nonanal is derived from the oxidation of oleic acid (C18:1*n*–9) as described in Belitz [36] and deMan [27]. This compound is perceived as fat, citrus, or green [23] (Flavornet database). In this study, its RPA value was 10.27%, ranking third in abundance after 2-propanone. The three aforementioned volatile compounds provided all together 57.17% of the total GC-MS detected compounds in the current study.

The effect of ageing on volatile compound percentage was statistically significant (*p* < 0.05) for tridecane, 3-methylbutanal, heptanal, 2-heptanone, 3-octanone, 1,4-dimethylbenzene, and 1-octen-3-ol (Table 1). Among these compounds, only 1,4-dimethylbenzene decreased with ageing. Tridecane showed a slight increase, whereas 3-methylbutanal, 2-heptanone, 3-octanone, and 1-octen-3-ol showed a high increase during ageing. This effect might have been pronounced in the present research because meat was aged under an oxygen-permeable film, thus the interaction between protein oxidation products and some amino acids might have been more pronounced [37]. No significant effect in the other identified volatile compounds percentage was observed with ageing (*p* > 0.05; Table 1).

Table 2 summarises some of the most recent and relevant papers on lamb volatile compounds. As can be observed, even if previous research was mainly carried out on animals older than the ones in the present study, there is a wide variety of coinciding volatile compounds that characterise lamb meat. Furthermore, none of the previous research addressed the effect of ageing. Elmore et al. [32] studied the volatile compound occurrence in Longissimus dorsi muscle of Suffolk cross breed lambs and reported 16 of the 26 volatile compounds identified in the present study (Table 2). Elmore et al. [32] fed lambs until approximately 40 kg LW with lipid supplements and the volatile compounds desorption was carried out at lower temperature (40 °C).

The studies of Vasta et al. [39] and Almela et al. [38] evaluated the volatile compounds in the Longissimus dorsi muscle at similar age/body weight as the present study, although in different breeds (“Leccese” and “Segureña”, respectively). They reported 11 and six volatile compounds in concordance with the present study, respectively (Table 2).

Bueno et al. [13] performed a study on the Longissimus thoracis et lumborum muscle of male lambs of Rasa Aragonesa breed slaughtered at 70 days (18–24 kg LW). The results showed that nine of the volatile compounds identified in the present study were also detected by Bueno et al. [13]. Moreover, Bravo-Lamas et al. [24] also observed nine volatile compounds coinciding with the ones obtained in the present study in lambs of the same commercial type.

Considering the experimental differences mentioned above, around 11 compounds were identified in most of the seven works presented. Thus, the following could be listed among lamb meat’s most characteristic volatile compounds: 2-methylpropanal, 2-methylbutanal, 3-methylbutanal, pentanal, hexanal, heptanal, octanal, nonanal, 2-heptanone, toluene, and 1-octen-3-ol.

Four of the compounds that were commonly identified in lamb meat (pentanal, hexanal, heptanal, and 1-octen-3-ol) may be derived from the decomposition of C18:2*n*–6 fatty acid (linoleic acid) [32]. This finding is in agreement with Arana et al. [41], who reported that animals from Rasa Aragonesa breed slaughtered at 26–28 kg live weight and fed a concentrate-based diet showed a high percentage of C18:2*n*–6 (8%), being Navarra and Rasa Aragonesa breeds from a common root.

In addition, 2-methylpropanal, 2-methylbutanal and 3-methylbutanal with a total RPA% of 2.68 are Strecker aldehydes that can originate from the catabolism of branched chain amino acids [42]. These compounds have low detection thresholds and distinctive odour characteristics as malty, pungent and sweet notes [25].

It has been postulated that octanal and nonanal (added RPA of 12.9% in this study) may have been formed from the oxidation of C18:1*n*–9 (oleic acid) [27,43]. Arana et al. [41] reported that animals from the Rasa Aragonesa breed of sheep, slaughtered at 26–28 kg live weight and fed a concentrate-based diet, showed a high percentage of C18:1*n*–9 (37%).

Finally, some aliphatic aldehydes and furans are reported to be formed by the oxidation of PUFA [44]. 2-penthylfuran, observed in three of the seven studies compiled in Table 2, is postulated to be a flavour compound derived from the oxidation of C18:3*n*–3 (linoleic acid) [27], giving herbal and legume notes to meat. Urrutia et al. [45] reported high percentages of C18:1*n*–9 (28%) in animals from the same breed and fed under similar conditions to the ones in the present study.

Bravo-Lamas et al. [24] reported that the odour profile of different Spanish light lambs was very similar, and thus, considering the volatile compound profile, lamb from the present study could be described tentatively as green, meaty, roasted, and fatty.

**Figure 1:**
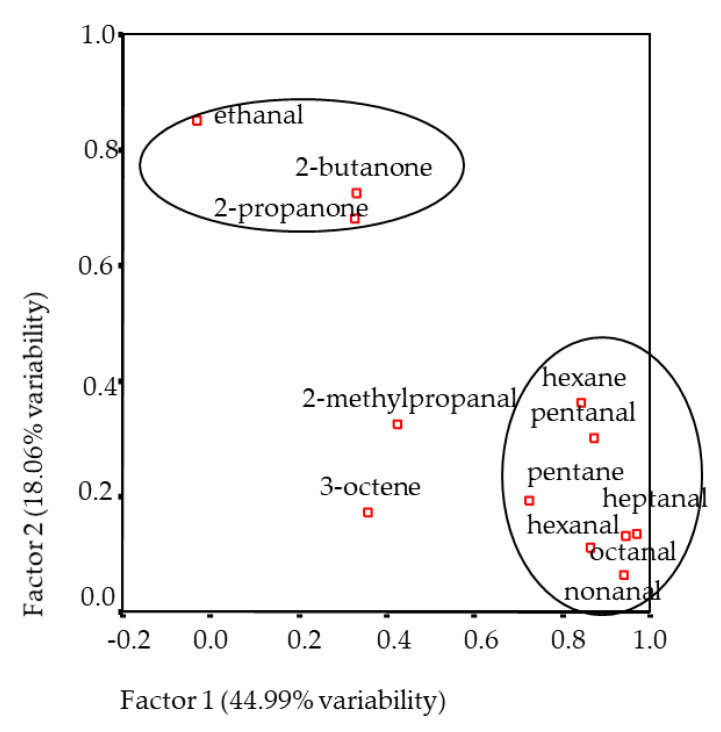
Plot of the volatile compounds from the headspace of the Longissimus thoracis muscle from Navarra breed lambs on the bidimensional space formed by factors 1 and 2.

To summarise, a multivariate PCA analysis was carried out with those volatile compounds present in all the studied samples: pentane, hexane, 3-octene, ethanal, 2-methyl-propanal, pentanal, hexanal, heptanal, octanal, nonanal, 2-propanone, and 2-butanone. In the factorial analysis, there were three factors obtained that explained 81.42% of total variability. Factor 1 (44.99%, Figure 1) was positively related with pentane, hexane, pentanal, hexanal, heptanal, octanal, and nonanal. The first factor could be named as an “oxidation” factor, as the aliphatic aldehydes represented are said to be formed by fatty acid oxidation [27]. Factor 2 (18.06%) was positively related with 2-propanone, 2-butanone, and ethanal. Factor 3 (13.38%) related negatively with 2-methyl-propanal and 3-octene.

### 3.2. Odour and Flavour Attributes

**Figure 2:**
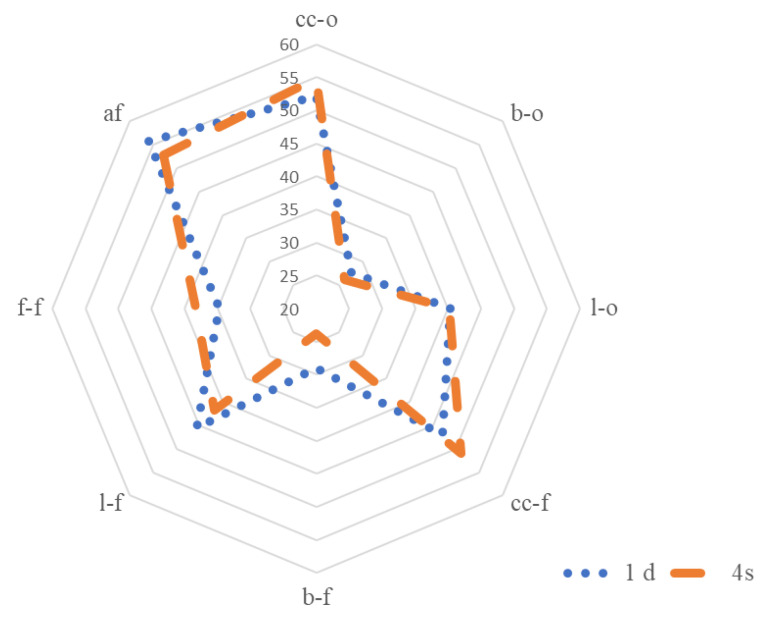
Odour and flavour attributes of the Longissimus thoracis muscle from Navarra breed lambs aged 1 and 4 days (cc-o: characteristic odour; b-o: blood odour; l-o: liver odour; cc-f: characteristic flavour; b-f: blood flavour; l-f: liver flavour; f-f: fatty flavour; af: aftertaste). Scale: 150-mm unstructured hedonic scale, with a mark 10 mm from the left, representing “low intensity” and 10 mm from the right, representing “high intensity”.

Meat from lambs of the Navarra breed in the current study was characterised by medium to low characteristic lamb and livery odour, characteristic lamb and livery flavour and aftertaste, a low bloody odour, and bloody and fatty flavour (Table 3, Figure 2). These low flavour and odour intensity scores might be attributed to the low intramuscular fat content of this meat. Urrutia et al. [45] reported an average intramuscular fat percentage of 3% for this sheep breed at this light commercial weight. Additionally, the low scores could be attributed to the short duration of the feeding period (around 40 days). This fact might have caused an insufficient accumulation of flavour and aroma compound precursors, as reported by Bravo-Lamas et al. [24]. Besides, this flavour profile was scarcely affected by ageing because this factor was only significant (*p* < 0.05) for bloody flavour, which decreased from day 1 to day 4. The rest of the sensory attributes showed no ageing effect (*p* > 0.05; Table 3). Nevertheless, the tendency, as shown in Figure 2, was that ageing increased lamb’s characteristic odour and flavour.

**Figure 3:**
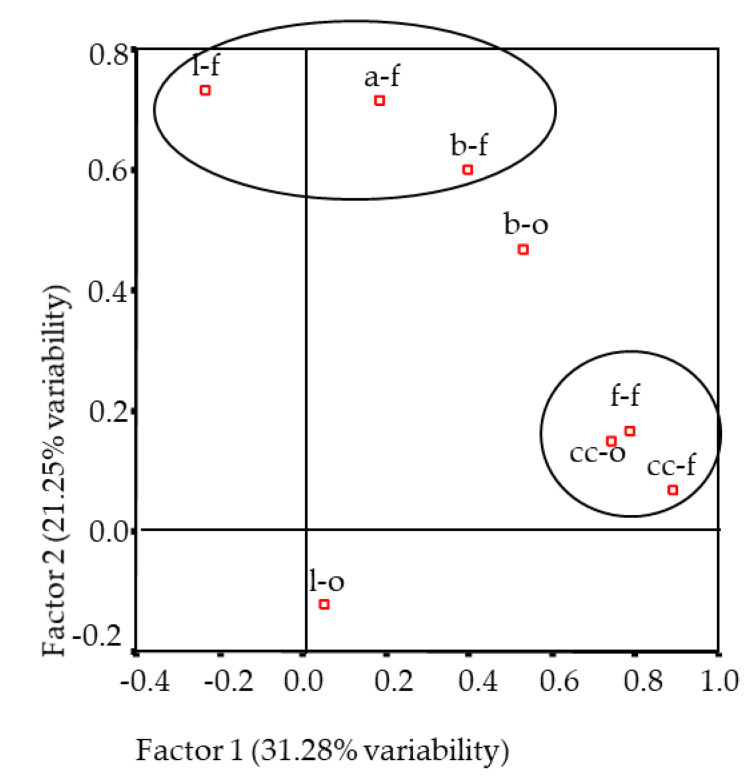
Plot of the the odour and flavour attributes of the Longissimus thoracis muscle from Navarra breed lambs on the bidimensional space formed by factors 1 and 2 (cc-o: characteristic odour; b-o: blood odour; l-o: liver odour; cc-f: characteristic flavour; b-f: blood flavour; l-f: liver flavour; f-f: fatty flavour; a-f: aftertaste).

Regarding the multivariate study, there were three principal components obtained that explained 69.93% of total variability at the PCA analysis. The first factor (31.28%, Figure 3) related to positively characteristic odour and flavour, and they were both related to fatty flavour. These results were in agreement with Pearson correlation coefficients between these attributes (*R*^2^ = 0.635, 0.577 and 0.684, respectively; *p* < 0.001).

The second factor (21.25%) related positively to bloody and livery flavour aftertaste, likely due to the presence of 2-propanone. In the present study, bloody and livery flavour did not show significant correlation coefficients (*p* > 0.05), but livery flavour showed a significant correlation with 2-propanone (*R*^2^ = 0.507, *p* < 0.001). The third factor (17.10%) was constituted only by livery odour.

### 3.3. Relationship between Volatile Compounds and Odour and Flavour Attributes

In spite of the tendencies observed regarding the sensory attributes and volatile compounds, no clear separation of days of ageing was observed from the abovementioned PCA analysis. Thus, in order to gain more knowledge, another multivariate analysis was carried out, gathering, on one side, the sensory attributes and, on the other, the volatile compounds detected in all the studied samples. Five factors that accounted for 78.24% of total variability were obtained. Only factors 3 (12.07%) and 4 (9.44%) related to sensory and instrumental (volatiles) values. Livery flavour was positively related to ethanal, 2-propanone and 2-butanone, and aftertaste was positively related to methylpropanal. This was also confirmed by the Pearson correlation analysis where only livery flavour showed significant correlation with ethanal (*R*^2^ = 0.353, *p* < 0.05), 2-propanone (*R*^2^ = 0.507, *p* < 0.01) and 2-butanone (*R*^2^ = 0.825, *p* < 0.05). These results are also in agreement with (Figure 1 and Figure 3), showing the relationship of the mentioned volatile compounds with livery flavour. Comparing the same (Figure 1 and Figure 3), the relationship between fat oxidation volatile compounds and characteristic odour and flavour and fatty flavour can also be inferred.

**Figure 4:**
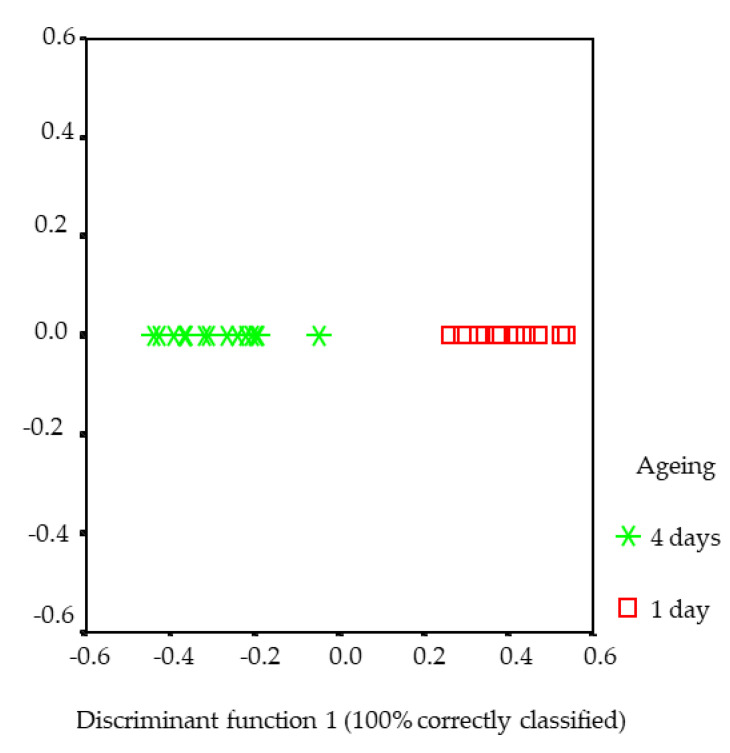
Separation of “days of ageing” groups obtained applying the discriminant analysis to odour and flavour attributes and volatile compounds of the Longissimus thoracis muscle from Navarra breed lambs.

Besides, and in order to infer which were the variables responsible for the changes between the days of storage, a discriminant analysis was carried out. In this analysis, 100% of the cases were correctly classified. This function separated day 1 in front of day 4 (Figure 4), showing that ageing was associated with a decrease in livery and bloody flavour, bloody odour and ethanal, and an increase in pentane, hexanal and heptanal. The latter showed a positive Pearson correlation with characteristic lamb odour (*R*^2^ = 0.319, *p* < 0.05) and were related to fresh green grass odour descriptors [24], even if lambs had been fed on concentrate, although the average time on feed was short (40 days).

Finally, in the present study, and despite the low statistical significance, it can be inferred from the discriminant analysis that ageing lamb from the Navarra breed might have a positive effect on meat sensory odour and flavour quality because there was an increase in characteristic lamb odour, related to a fresh green grass odour perception, and a decrease in livery and bloody flavour as well as bloody odour and aftertaste.

## 4. Conclusions

The greater occurrence of aldehydes, which are representative of the volatile profile of ruminant meat, is related to a high level of concentrate-rate in the diet of lambs, leading to an odour profile described as green grass, meaty, roasted, and fatty. Additional days on feed may result in an increase in the observed sensory attributes.

The effect of ageing was significant but not intense, probably due to the low intramuscular fat content of light lambs, a short time on feed, and a short ageing period (4 days), which was not enough to accumulate abundant flavour and aroma compounds. In any case, bloody flavour decreased from day 1 to day 4, and the trend was that ageing increased characteristic lamb odour and flavour. Consequently, ageing lamb from the Navarra breed does have a potentially positive effect on meat sensory odour and flavour qualities. In future research, carrying out a consumer panel might be helpful to confirm these findings.

## Figures and Tables

**Table 1:** Volatile compounds detected in the headspace of the Longissimus thoracis muscle from Navarra breed lambs.

Volatile Compounds	RT (min)	Method of Identification			RPA (%)	Ageing			*p*-Value	Odour Descriptors *
		LRI	RI	MS		1 Day	4 Days	SEM		
Aliphatic hydrocarbons										
Pentane	4.172	500			7.58	241.32	248.87	5.997	0.884	Alkane ^1^
Hexane	6.885	594	+	+	0.91	29.96	29.06	1.217	0.865	Alkane ^1^
3-octene	21.903	800	+	+	1.02	33.95	32.41	1.209	0.710	Sweet ^2^
2-octene	2.306	809	+	+	0.19	4.62	7.64	0.820	0.074	Alkane ^1^
2.2.4.6.6-pentamethylheptane	28.667	985	+	+	1.31	44.79	29.28	1.550	0.162	
Tridecane	35.708	1244		+	0.26	6.57	10.01	0.812	0.039	
Aliphatic aldehydes										
Ethanal	3.123	427		+	3.99	134.27	124.52	1.943	0.598	Pungent, ether ^1^
2-methyl propanal	5.566	548	+	+	1.32	39.45	46.08	1.658	0.425	Pungent, malt, green ^3^
3-methylbutanal	10.238	641	+	+	0.85	14.48	34.16	2.281	0.016	Malt ^3^, cocoa, almond ^1^
2-methylbutanal	10.988	651	+	+	0.51	8.86	18.10	2.068	0.180	Roasted, fermented ^2^; malty, pungent ^1^^,3^
Pentanal	14.08	692	+	+	1.79	58.00	58.12	2.116	0.993	Acrid, pungent ^2^
Hexanal	21.584	795	+	+	31.31	987.83	1039.64	13.557	0.741	Fresh grass, green grass ^2^, grass, tallow, fat ^1^
Heptanal	25.849	899	+	+	2.19	56.68	86.97	2.452	0.050	Green, floral ^2^, fat, citrus, rancid ^1^
Octanal	28.964	1002	+	+	2.63	75.78	95.03	2.145	0.121	Orange peel ^2^, fat, soap, lemon, green ^1^
Nonanal	31.547	1097	+	+	10.27	301.92	362.00	4.920	0.210	Fat, citrus, green ^1^
Aliphatic ketones										
2-propanone	43.225	505	+	+	15.59	593.90	433.13	13.705	0.173	Fat ^4^
2-butanone	7.102	600	+	+	1.53	49.56	49.34	1.298	0.969	
2-heptanone	25.652	891	+	+	0.53	7.60	23.23	1.167	0.000	Musty, spicy ^2^, soap ^1^
3-octanone	28.484	980	+	+	1.01	20.14	43.00	1.453	0.000	
Aromatic hydrocarbons										
Benzene	10.723	648	+	+	0.63	15,56	28.92	2.390	0.069	
Toluene	19.828	771	+	+	0.53	29.21	11.64	1.888	0.142	Paint ^1^
1.4-dimethylbenzene	24.748	869	+	+	0.25	11.65	6.64	1.180	0.002	
Sulphur compounds										
Carbon disulphide	5.049	530	+	+	4.57	152.90	143.74	4.970	0.824	
Aliphatic alcohols										
Metanethiol	3.318			+	1.71	53.08	57.02	0.833	0.579	
1-octen-3-ol	28.304	977	+	+	4.79	63.89	241.23	4.233	0.000	Mushroom, damp, soil ^2^
Furans										
2-penthylfuran	28.651	985	+	+	2.33	58.13	76.54	3.103	0.460	Herbal, legume ^5^

RT: retention time (min). LRI: linear retention index as determined using n-alkanes on an HP-5 fused silica column under the same GS-MS conditions of the experiment. RI: retention index. Approximate identification by comparing the retention index with literature values [22]. MS: tentative mass spectrometry identification. Tentatively identified by matching the sample spectrum against the Wiley 275 library spectrum. RPA: relative percentage abundance. Values are expressed in area units (×10^5^). * Odour descriptors: ^1^ Flavornet database [23]; ^2^ Bravo-Lamas et al. [24]; ^3^ Madruga et al. [25]; ^4^ Mottram, [26]; ^5^ deMan [27].

**Table 2:** Review of volatile compounds in lamb meat.

Volatile Compounds Detected at the Present Study	Elmore et al. [32]	Almela et al. [38]	Vasta et al. [39]	Bueno et al. [13]	Bravo Lamas et al. [24]	Karabagias [40]	Gkarane et al. [33]
Pentane							
Hexane							
3-octene							
2-octene							
2,2,4,6,6-pentamethylheptane	+					+	
Tridecane	+						+
Ethanal							
2-methyl propanal	+		+	+			
3-methylbutanal	+			+			+
2-methylbutanal	+			+	+		+
Pentanal	+	+	+	+	+		+
Hexanal	+	+	+	+	+	+	+
Heptanal	+	+	+	+	+	+	+
Octanal	+	+	+	+	+	+	+
Nonanal	+	+	+	+		+	+
2-propanone							
2-Butanone	+		+		+		
2-heptanone	+		+	+	+		+
3-octanone						+	
Benzene	+						
Toluene	+		+			+	+
1,4-dimethylbenzene							
Carbon disulphide			+		+	+	
Metanethiol							
1-octen-3-ol	+	+			+	+	+
2-penthylfuran	+		+				+
Total number of volatile components identified	111	35	37	33	43	17	64
Sheep Breed	Suffolk-cross	Segureña	Leccese	Rasa Aragonesa	−^1^	Fries–Arta	Texel × Scottish Blackface
Sex	−^1^	−^1^	Male	Male	−^1^	Male	Male
Live weight/age	40 kg	25 kg	45 days	70 days	−^1^	3 months	248–273 days
Studied muscle	LD	LD	LD	LD	LD	Leg (hind shank)	LD
Extraction method	Headspace	SPME	SPME	Vac elut cartbridges	Dynamic headspace	Headspace	SPME

LD = Longissimus dorsi. SPME = Solid-Phase Microextraction. ^1^ The “−“ sign indicates not reported data.

**Table 3:** Mean scores of sensory odour and flavour attributes of the Longissimus thoracis muscle from Navarra breed lambs aged 1 and 4 days.

Sensory Attributes	Ageing ^1^			
	1 Day	4 Days	SEM	*p*-Value
Odour				
Characteristic Lamb Flavour	52.18	54.85	0.371	0.433
Bloody	27.62	26.07	0.255	0.511
Livery	40.32	40.22	0.250	0.965
Flavour				
Characteristic Lamb Flavour	47.06	51.08	0.394	0.267
Bloody	29.05	23.78	0.285	0.045
Livery	45.60	41.79	0.372	0.265
Fatty	34.74	38.21	0.349	0.280
Aftertaste	55.83	52.75	0.388	0.389

^1^ Scale: 150-mm unstructured hedonic scale, with a mark 10 mm from the left, representing “low intensity” and 10 mm from the right, representing “high intensity”.

## Data Availability

Not applicable.
